# The Value of Case Reports in Systematic Reviews from Rare Diseases. The Example of Enzyme Replacement Therapy (ERT) in Patients with Mucopolysaccharidosis Type II (MPS-II)

**DOI:** 10.3390/ijerph17186590

**Published:** 2020-09-10

**Authors:** Miguel Sampayo-Cordero, Bernat Miguel-Huguet, Andrea Malfettone, José Manuel Pérez-García, Antonio Llombart-Cussac, Javier Cortés, Almudena Pardo, Jordi Pérez-López

**Affiliations:** 1 Medica Scientia Innovation Research (MedSIR), Ridgewood, NJ 07450, USA; andrea.malfettone@medsir.org (A.M.); jose.perez@medsir.org (J.M.P.-G.); antonio.llombart@medsir.org (A.L.-C.); jacortes@vhio.net (J.C.); 2 Medica Scientia Innovation Research (MedSIR), 08018 Barcelona, Spain; 3 Department of Surgery, Hospital de Bellvitge, L’Hospitalet de Llobregat, 08907 Barcelona, Spain; hanscastorp77@gmail.com; 4 Institute of Breast Cancer, Quiron Group, 08023 Barcelona, Spain; 5 Hospital Arnau de Vilanova, Universidad Católica de Valencia “San Vicente Mártir”, 46015 Valencia, Spain; 6 Vall d’Hebron Institute of Oncology (VHIO), 08035 Barcelona, Spain; 7 Albiotech Consultores y Redacción Científica S.L., 28035 Madrid, Spain; a.pardo@albiotech.com; 8 Department of Internal Medicine, Hospital Vall d’Hebron, 08035 Barcelona, Spain; jordiperezlopez.eemm@gmail.com

**Keywords:** systematic review, meta-analysis, randomized clinical trial, nonrandomized study, case report, mucopolysaccharidosis, enzyme replacement therapy, rare disease, clinical studies

## Abstract

Background: Case reports are usually excluded from systematic reviews. Patients with rare diseases are more dependent on novel individualized strategies than patients with common diseases. We reviewed and summarized the novelties reported by case reports in mucopolysaccharidosis type II (MPS-II) patients treated with enzyme replacement therapy (ERT). Methods: We selected the case reports included in a previous meta-analysis of patients with MPS-II treated with ERT. Later clinical studies evaluating the same topic of those case reports were reported. Our primary aim was to summarize novelties reported in previous case reports. Secondary objectives analyzed the number of novelties evaluated in subsequent clinical studies and the time elapsed between the publication of the case report to the publication of the clinical study. Results: We identified 11 innovative proposals in case reports that had not been previously considered in clinical studies. Only two (18.2%) were analyzed in subsequent nonrandomized cohort studies. The other nine novelties (81.8%) were analyzed in later case reports (five) or were not included in ulterior studies (four) after more than five years from their first publication. Conclusions: Case reports should be included in systematic reviews of rare disease to obtain a comprehensive summary of the state of research and offer valuable information for healthcare practitioners.

## 1. Introduction

Rare diseases and ultra-rare diseases are defined by a prevalence of ≤50 patients and ≤1 patient per 100,000 people, respectively. This low prevalence of such diseases makes the conduction of randomized and prospectively designed studies very hard [1,2]. As an example, mucopolysaccharidosis type II (MPS-II) has a prevalence of 1 in 140,000–156,000 patients [3]. In this case, the registries of clinical cases usually are the best option for clinical research [4,5]. Furthermore, in rare diseases, the phenotype and genotype heterogeneity limit the generalization of the data from clinical trials to clinical practice [1,6]. Thus, pharmacological treatment of patients with orphan and ultra-orphan drugs is more dependent on individualized strategies than treatment of more prevalent diseases [7,8].

The value of case reports as individual studies has been stated in previous reviews [9]. Investigators have considered that case reports are appropriate to report new clinical findings, generating new hypothesis about pharmacological therapies, educational value, and high applicability in situations where other designs are not feasible. However, case reports are limited by their retrospective, nonblinded, and nonrandomized trial design, constituting a source of biases that could affect the study outcome. Therefore, findings provided by case reports might not be generalizable and could not be useful to establish a cause–effect relationship, with a consequent high risk of over-interpretation [9,10,11]. Accordingly, it is uncommon to include case reports in systematic reviews and meta-analyses [12,13].

Systematic reviews were developed to ensure that decisions affecting human health would be informed with the complete understanding of the most relevant research evidence. Thus, the purpose of a systematic review is to provide a summary of the state of research knowledge on an intervention, diagnostic test, prognostic factor, or other health or healthcare topics [12]. It has been suggested that novelties about available interventions are published in case reports five or more years earlier than in clinical studies [13]. Accordingly, a detailed review of a health topic should not exclude published clinical reports, especially in the field of rare diseases, where the individualization of treatments or management strategies has a great relevance on clinical practice [14,15].

A systematic review evaluating the efficacy and safety of enzyme replacement therapy (ERT) for treatment of patients with MPS-II has been previously published [2]. The aim of the present study was to review the novelties on outcomes or therapeutic strategies reported in case studies of the mentioned systematic review [2]. Secondary objectives included the number of novelties evaluated in subsequent clinical studies and the time elapsed between the publication of a novelty in a case report and its subsequent inclusion in a clinical study. Consequently, we searched for the latest clinical studies that incorporated the research questions stated by previous case reports.

## 2. Methods

### 2.1. Data Sources, Study Selection, and Quality Assessment from the Previous Meta-Analysis of Case Reports

The case reports selected were previously included in a recent meta-analysis by Sampayo-Cordero M. et al. [2]. The systematic review included case reports of MPS-II patients who were treated with ERT, and the search was carried out in Excerpta Medica Database (EMBASE), Medical Literature Analysis and Retrieval System Online (MEDLINE), The Cochrane Library (Cochrane Database of Systematic Reviews, Cochrane Central Register of Controlled Trials, and Cochrane Methodology Register and Health Technology Assessment Databases), as well as on the Latin American and Caribbean Literature on Health Sciences (LILACS). The search methods, study selection, and quality assessment are deeply described in the mentioned publication [2]. All case reports published up to 31 December 2015 were included in the search. For the current study, we selected all case reports that evaluated a new outcome or a new therapeutic strategy with ERT in MPS-II patients. Case reports excluded objectives that were similar to those described in previous randomized and nonrandomized studies [16,17,18,19,20].

### 2.2. Data Search of New Clinical Studies

The case reports selected from the previous meta-analysis were used to search new clinical studies with the same issue. We searched for the most recent clinical studies that incorporated the novelties proposed by case reports in patients with MPS-II treated with ERT. We searched MEDLINE and GOOGLE (all) for case reports that reported these novelties and retrieved the citations included in sections “cited by” or “comment”. There was no restriction of dose, treatment duration, administration via (intravenous or intrathecal), type of study design, or language. All published studies up to 1 July 2020 were included in our comprehensive search.

### 2.3. Study Selection

We selected all clinical studies citing the original case report, which accomplished the following selection criteria:

Inclusion criteria:

(1) Clinical studies (randomized or nonrandomized) and case reports of patients with MPS-II treated with ERT.
(2) The study design evaluates as primary, secondary, or exploratory objective the novelty proposed by the case report.
(3) The results reported included data from new patients where the novelty was the object of research.

Exclusion criteria:

(1) Clinical studies or case reports in other diseases or based on treatment without ERT.
(2) Systematic or literature reviews that do not include analyses of new patients.

### 2.4. Quality Assessment

The study was prospectively designed to select the studies that analyzed the novelties proposed by previous case reports on MPS-II. Results of current meta-analysis are reported in accordance with the Preferred Reporting Items for Systematic Reviews and Meta-Analysis (PRISMA) and Meta-Analyses and Systematic Reviews of Observational Studies (MOOSE) guidelines [21,22]. Three investigators (M.S.-C., B.M.-H., and A.P) entered findings into a database, independently reviewed citations/abstracts from the database, and hand searched and selected full relevant articles and documents for data extraction using preset criteria. Discrepancies were resolved through discussion or input from a fourth reviewer (J.P.-L.).

### 2.5. Primary Outcome

The primary outcome was a synthetic description of novelties proposed by the case reports that were selected in the meta-analysis of Sampayo-Cordero M. et al. [2]. We compared the objectives and ERT schedules of each selected case report with those of clinical studies published prior to the meta-analysis [16,17,18,19,20]. The case reports with objectives or therapy strategies equal to those of the clinical studies were not considered. Only the case reports analyzing novel objectives and therapy strategies were described.

### 2.6. Secondary Outcomes

A. The number of novelties proposed by prior case reports evaluated in subsequent clinical studies. The subsequent clinical studies were reported by type of study (randomized, cohort study, and case report).

B. The time between the publication of the case report and the clinical study.

### 2.7. Statistical Methods

Novelties described in each case report were summarized in a narrative format. The number of novelties and clinical studies was reported as number and percentage. The time elapsed between the publication of a case report including a specific novelty and the follow-up clinical study or studies was calculated subtracting the date of the first clinical study to the date of the first case report analyzing such novelty. If there was not a clinical study analyzing that novelty, the cutoff date was 1 July 2020.

## 3. Results

### 3.1. Data Search Results

An accurate selection of case reports with novelties published by Sampayo-Cordero et al. [2], which included publications through 31 December 2015, identified 27 citations [2]. Of 27 studies identified, 11 were excluded because they evaluated objectives and ERT schedules equivalent to those of previous clinical studies [16,17,18,19,20]. Thus, 16 publications of case reports were included in the study and their summary identified 11 different novelty proposals about ERT in MPS-II patients (primary objective).

**Figure 1:**
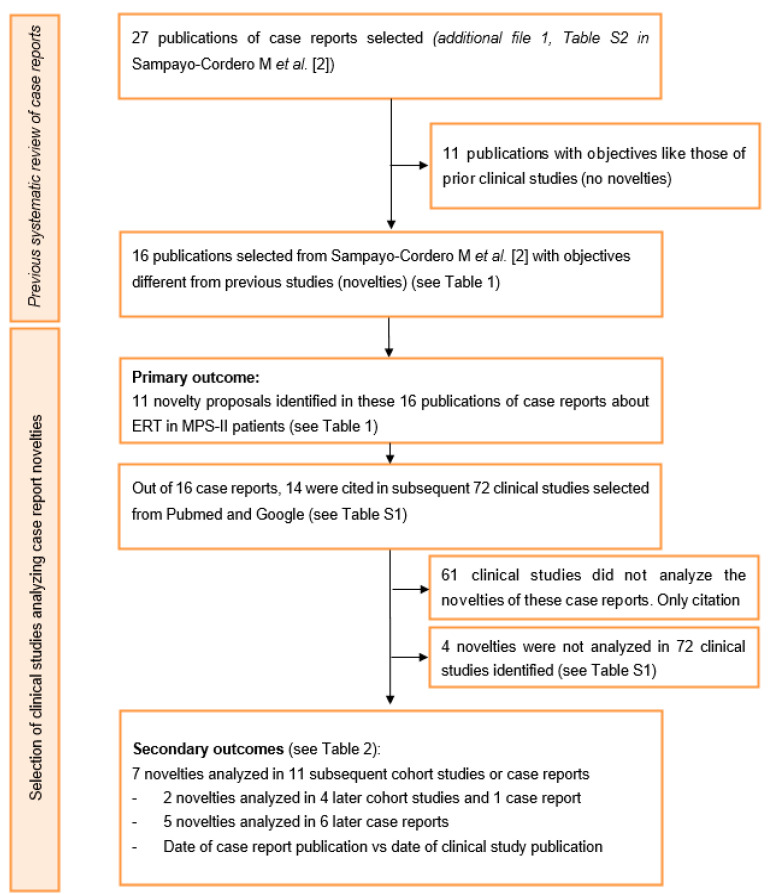
Flow of case reports that evaluated the efficacy and safety of enzyme replacement therapies in mucopolysaccharidosis type II (<2016). ERT—Enzyme replacement therapy; MPS—Mucopolysaccharidosis.

Searching for these 16 case reports, we identified 72 following publications. However, 11 of 72 publications analyzed the novelties of these case reports, while the remaining 61 publications were excluded because they did not (see Supplementary Table S1 and Figure 1).

### 3.2. Primary Outcome

Our study identified 11 innovative proposals in case reports that had not been previously considered in clinical studies. Most of them evaluated the efficacy of ERT in different outcomes and therapeutic situations compared with those included in previous clinical studies. They considered skin lesions, hyperactivity, aggressive behavior, language functioning, social interaction, central nervous system development, epileptogenic symptoms, effects in vision, and bone abnormalities. In addition, some case reports evaluated the efficacy of ERT in patients with physical abnormalities or affected by hematological diseases. Finally, three studies suggested new therapeutic strategies to increase the immunotolerance for ERT and to reduce dermatan sulfate in plasma (see Table 1) [23,24,25].

### 3.3. Secondary Outcomes

Of 11 novelties, only two (18.2%) were analyzed in subsequent cohort studies: (1) The efficacy of early ERT in central nervous system and (2) bone abnormalities. The other nine novelties (81.8%) were analyzed in later case reports (five novelties) or were not included in ulterior studies (four novelties). The five novelties evaluated in new case reports were: (1) The immune tolerance regimen, (2) the effect of ERT in skin, (3) the effect of ERT in epileptogenic symptoms, (4) the effect of ERT in ocular function, and (5) the effect of ERT in hematological diseases. So, novelties proposed in case reports are usually confirmed with later case reports. None of the proposed innovations was included in the objectives of a subsequent randomized clinical trial. In most cases, novel strategies suggested in case reports were not evaluated in a cohort or randomized study after 5 to 12 years (see Table 2).

## 4. Discussion

In the last years, the interest in case reports by clinical research community as well as the number of journals that publish case reports have increased rapidly [61]. Clinical cases’ journals have been included in the Medline database for medical journals and registries of case reports have been developed [9]. The greatest advantages of clinical cases are represented by their ability to detect novelties, the generation of new hypotheses, and their usefulness in the investigation of rare diseases [9]. Additionally, the individualization of therapies and management strategies is a key factor to successfully treat patients with rare diseases. So, new proposals dealing with uncommon situations in the treatment of rare-disease patients have great relevance to translate clinical research into clinical practice [7,8]. One of the aims of systematic reviews on therapeutic interventions is to summarize the state of art in this intervention [12]. However, case reports are systematically excluded in systematic reviews in rare-disease research, especially when other designs with a higher level of evidence are included [12,15,62,63,64,65]. Therefore, important innovations, which could contribute to a better management of the patient treated with orphan drugs, are systematically excluded from systematic reviews of rare diseases [2,13].

Our results on MPS-II patients treated with ERT showed that case reports reported findings relevant to complement patient management. The efficacy of ERT was explored on key outcomes (social behavior, skin lesions, central nervous system development, bone abnormalities, visual acuity) and specific situations (serious respiratory impairment, hematological disease, and physical abnormalities) that had not been considered in previous clinical studies. Additionally, case reports also proposed new therapeutic strategies to deal with immune reactions against ERT [23,24,25,26,27,28,29,30,31,32,33,34,35,36,37,38,49]. In accordance with previous results, we observed that most of these novelties were not assessed in subsequent clinical studies after five years from the publication of the case report [13]. Importantly, the new studies assessing these proposals were usually case reports [31,50,51,57,59,60]. Therefore, the evidence and innovations provided by case reports will take a long time to be incorporated into clinical studies on rare diseases [13].

Some previous systematic reviews in MPS have included case reports with clinical studies [2,13,14,15]. As we observed in our results, the inclusion of case reports was necessary to complete the evaluation of the ERT profile in specific situations (pregnancy, ERT discontinuation, surgery interventions, recovering from cerebral infarctions, serious respiratory impairment, or hematological diseases), key alternative outcomes (frequency of respiratory infections, texture of hair and/or skin, skeletal disease, or disease-related hospitalizations), and alternative therapeutic strategies with ERT [2,13,14,15]. Additionally, the inclusion of case reports allows us to assess the heterogeneity between the patients included in clinical studies and those from clinical cases; and it is useful to evaluate if ERT efficacy and safety results are equivalent among different study designs. The inclusion of case reports allows including all available evidence on ERT and it could increase the power of analyses performed [2,13,14,15]. We observed in previous systematic reviews including case reports that they used two alternative strategies. The first proposal summarized the results of the case reports and discussed them qualitatively. The results from case reports were not included in meta-analyses [14,15]. The second method aggregated the results of case reports in a meta-analysis. The strategy to analyze the results was to define two improvement (or impairment) criteria. One for individual patients included in the meta-analyses (e.g., increase in 6-min walk test over the baseline assessment) and another for the specific outcome or group of patients evaluated (e.g., a significant difference versus a 5% null hypothesis in the rate of patients with improvement in the walk test) [2,13]. Both methodologies represent an advantage with respect to excluding clinical cases from systematic reviews, since they allow enriching the study’s results and conclusions [2,13,14,15]. Importantly, these studies have demonstrated that standardization and a good definition of outcomes evaluated in case reports increase the validity of their results [2,13]. Therefore, the tools to assess the quality of case reports and case series are very relevant in systematic reviews that include clinical cases [66]. Different efforts have been done to homogenize and upgrade the quality of case reports [67] or to search for case reports in clinical databases [9,68].

Previous studies and guidelines have considered that clinical cases could be included in systematic reviews [2,13,69]. However, they do not consider the exclusion of case reports a serious concern if there are other designs available. Case report exclusion from systematic reviews in rare diseases has a greater impact than just the loss of a few cases with rare conditions or rare adverse events [12,69]. In fact, our analyses show that most of the novelties proposed in case reports are not subsequently contrasted in clinical studies with more patients (after 5 to 12 years), which might be due to the case reports being overlooked, since they are not included in reviews.

Our study took advantage of a previous systematic review to select the individual case reports analyzed in this communication. We considered that this strategy provides an important quality control measure. The selection procedures was previously accepted after a peer review revision [2].

An important point in our strategy for data search was retrieving articles, which cited selected case reports. Some articles could have analyzed novelty of a case report without citing it. However, in rare diseases, and in MPS-II disease specifically, the number of clinical studies is quite limited, so it is not likely to happen in many communications [62]. In addition, our study only included case reports from MPS-II patients. Therefore, it is not clear whether the results obtained in this study could be extrapolated to other rare diseases, although it is important to note that the methods for aggregating clinical cases obtained similar results in different MPS, namely MPS-I and MPS-II [2,13,14,15].

It is usually considered that a MEDLINE search alone is not adequate for answering relevant questions [12]. In accordance, we have increased the scope of our search with GOOGLE searcher, since it is commonly used in all types of searches and has specific tools for systematic reviews and scientific searches [70,71].

## 5. Conclusions

Different authors have underlined the impact of individual clinical reports’ results in clinical practice and research [11,72]. Previous studies and guidelines have suggested the inclusion of case reports in systematic reviews, but they did not consider the exclusion of case reports a serious concern if there are other designs available with a higher grade of evidence than case reports [12,69]. However, according to our results, most of the novel procedures proposed in case reports are not evaluated in later clinical studies. Thus, case reports should be included in systematic reviews of rare diseases to make a complete summary of the state of research, since excluding relevant novelties proposed in case reports could reduce the usefulness of the review for clinical practice.

## 6. Patents

A.L.-C. declares the following stock or other ownership: Medica Scientia Innovation Research (MedSIR) and Initia-Research. J.C. declares the following stock, patents, and intellectual property: MedSIR.

## Figures and Tables

**Table 1:** Novelties proposed in case reports for enzyme replacement therapy in mucopolysaccharidosis type II patients.

Novelties Proposed in Case Reports for Treatment of MPS-II Patient with ERT *	Case Reports Selected
**New therapeutic strategies to increase the immunotolerance for ERT**	
(1) Immune tolerance regimen and desensitization procedures	Kim et al. 2014; Gkavogiannakis N et al. 2015 [23,24]
(2) New therapeutic strategies based on the evaluation of plasmatic dermatan sulfate	Volpi et al. 2013 [25]
**Different outcomes and therapeutic situations**	
(3) Effects in pebbling skin lesions	NoH et al. 2014; Marín LL et al. 2012 [26,27]
(4) Effects in hyperactivity, aggressive behavior, language functioning, and social interaction	Puiu M et al. 2013 [28]
(5) Evaluation of the effects of ERT in central nervous system development	Wang RY et al. 2009 [29]
(6) Evaluation of the effects of ERT in epileptogenic symptoms	Kinoshita M et al. 2014; Bonanni P et al. 2012 [30,31]
(7) ERT effects in vision	Sanchez JI et al. 2015; Lau HA et al. 2015 [32,33]
(8) ERT effect in autoimmune anemia, thrombocytopenia, or thrombocytopenic purpura	Fisher et al. 2015; Uz B et al. 2012 [34,35]
(9) Botulinum Toxin for the treatment of equinus deformity with an ERT	Nava E et al. 2012 [36]
(10) ERT effects in involuntary movements (chorea)	Farooq MU et al. 2008 [37]
(11) Early ERT effects in bone abnormalities	Papadia F et al. 2011 [38]

ERT—enzyme replacement therapy, MPS-II—mucopolysaccharidosis type II. *—Only case reports reporting novelties were analyzed. Case reports excluded analyzed objectives similar to those described in previous randomized and nonrandomized studies (Muenzer et al. 2006, Muenzer et al. 2011, Tolar J. et al. 2008, Wynn F. et al. 2009, and Eisengart J.B. et al. 2013) [16,17,18,19,20]. The case reports excluded were: Lampe et al. 2014, Biviana et al. 2014, Christiano et al. 2013, Sato et al. 2013, Tajima et al. 2013, Hoffmann B. et al. 2011, Tylki-Szymanska et al. 2012, Pérez-Calvo et al. 2011, Tchan M.C. et al. 2011, Westhoff M. et al. 2011, and Galán Gómez E. et al. 2008 [39,40,41,42,43,44,45,46,47,48,49].

**Table 2:** Clinical studies evaluating novelties proposed by prior case reports.

Novelties Proposed in Case Reports for ERT in MPS-II Patients (Citation) *	Next Studies, Article Type (Citation)	Time Until Clinical Study (Case Reports not Considered)
(1) Immune tolerance regimen and desensitization procedures [23,24]	**Case report** (Julien DC et al., 2020) [50]	No clinical study after 6 years
(2) New therapeutic strategies based on the evaluation of plasmatic dermatan sulfate [25]	No new citations	No clinical study after 7 years
(3) Effects in pebbling skin lesions [26,27]	**Case report** (Srinivas SM et al., 2017) [51]	No clinical study after 8 years
(4) Effects in hyperactivity, aggressive behavior, language functioning, and social interaction [28]	No clinical study after 7 years	No clinical study after 7 years
(5) Evaluation of the effects of ERT in central nervous system development [29]	**Cohort studies** Matsubara et al., 2017; Manara et al., 2011 **; Yund et al., 2015; Tanaka A et al., 2012 (**3 case reports**) Crowe et al., 2017 [52,53,54,55,56]	2 years/Not randomized
(6) Evaluation of the effects of ERT in epileptogenic symptoms [30,31]	**Case report** Scarpa et al., 2017 Bonanni et al., 2014 [57,58]	No clinical study after 8 years
(7) ERT effects in vision [32,33]	**Case report** (Yamanishi R et al., 2019) [59]	No clinical study after 5 years
(8) ERT effect in autoimmune anemia, thrombocytopenia, or thrombocytopenic purpura [34,35]	**Case report** (Alcántara-Ortigoza et al., 2016) [60]	No clinical study after 8 years
(9) Botulinum Toxin for the treatment of equinus deformity with an ERT [36]	No new citations	No clinical study after 8 years
(10) ERT effects in involuntary movements (chorea) [37]	No new citations	No prospective study after 12 years
(11) Early ERT effects in bone abnormalities [38]	**Cohort study** (Manara R et al., 2011 **) [53]	4 months/Not randomized

ERT: Enzyme replacement therapy, MPS-II: Mucopolysaccharidosis type II. * Only case reports reporting novelties were analyzed. Case reports excluded analyzed objectives similar to those described in previous randomized and nonrandomized trials (Muenzer et al. 2006, Muenzer et al. 2011, Tolar J. et al. 2008, Wynn F. et al. 2009, and Eisengart J.B. et al. 2013 [16,17,18,19,20]). The case reports excluded were: Lampe et al. 2014, Biviana et al. 2014, Christiano et al. 2013, Sato et al. 2013, Tajima et al. 2013, Hoffmann B. et al. 2011, Tylki-Szymanska et al. 2012, Pérez-Calvo et al. 2011, Tchan M.C. et al. 2011, Westhoff M. et al. 2011, and Galán Gómez E. et al. 2008 [39,40,41,42,43,44,45,46,47,48,49]. ** This study analyzed two novelties proposed by previous case reports.

## Supplementary Materials

The following are available online at https://www.mdpi.com/1660-4601/17/18/6590/s1: Supplementary Table S1. Novelties proposed in case reports of ERT in MPS-II patients and in clinical studies citing these case reports. References [16,17,18,19,20,23,24,25,26,27,28,29,30,31,32,33,34,35,36,39,40,41,42,43,44,45,46,47,48,49,50,51,52,53,54,55,56,57,58,59,60,73,74,75,76,77,78,79,80,81,82,83,84,85,86,87,88,89,90,91,92,93,94,95,96,97,98,99,100,101,102,103,104,105,106,107,108,109,110,111,112,113,114,115,116,117,118,119,120,121,122,123,124,125,126,127,128,129,130,131,132] are cited in the supplementary materials.
