# Supplementary material for: The Value of Case Reports in Systematic Reviews from Rare Diseases. The Example of Enzyme Replacement Therapy (ERT) in Patients with Mucopolysaccharidosis Type II (MPS-II)
Source: Int J Environ Res Public Health. 2020 Sep 10;17(18):6590. doi: 10.3390/ijerph17186590 (PMC7558586; doi:10.3390/ijerph17186590)
Supplement: Supplementary file 1 [file ijerph-17-06590-s001.pdf]

International Journal of *Environmental Research and Public Health*

MDPI

Supplementary Table S1. Novelties proposed in case reports for ERT in MPS II patients and clinical studies citing these case reports.

Case Reports Selected in Meta-Analyses	Novelties Proposed in Case Reports for ERT in MPS II Patients *	Next Studies about this Novelty (Article Type)	Articles Citing Previous Case Reports—Without New Patient Data in MPS II Patients Treated with ERT
**New therapeutic strategies to increase the immunotolerance for ERT**			
Kim et al. 2014 Gkavogiannakis N et al. 2015 [23,24]	(1) Immune tolerance regimen or desensitization procedure to ERT	Julien DC et al., 2020 [50] (Case report)	Parini R et al. 2020, Chen HH et al. 2019, Sawamoto K et al. 2019, Akyol MU et al. 2019, Lagler FB 2018, Lagler FB 2019 [73–78]
Volpi et al. 2013 [25]	(2) New therapeutical strategies based on the presence of of a discrete amount of plasmatic derman sulfate after 10 months of ERT	No new citations	
**Different outcomes and therapeutic situations**			
NoH et al. 2014; Marín LL et al. 2012 [26,27]	(3) Pebbling skin lesions decrease after ERT	Srinivas SM et al., 2017 [51] (Case report)	Singh A et al. 2020, Kubaski F et al. 2020, Tjarks et al. 2019 [79–81]
Puiu M et al. 2013 [28]	(4) ERT effects in hyperactivity, aggressive behaviour, languaje functioning, and social interaction	No new citations	
Wang RY et al. 2009 [29]	(5) Evaluation of the effects of ERT in central nervous system	(Cohort studies) Matsubara et al., 2017 Manara et al., 2011 ** Yund et al., 2015 Tanaka A et al. 2012, (3 case reports) Crowe et al., 2017 [52–56]	Almassi y Algahim, 2013; Valayannopoulos y Wijburg, 2011; D'Aco et al., 2012; Zafeiriou y Batzios, 2013; de Ruijter et al., 2012; Lin et al., 2013; Baldo et al., 2013; Higuchi et al., 2012; I. Dickson y H. Chen, 2011; Reichert et al., 2016; Yang y Prabhu, 2014; Calleja Gero et al., 2012; Coppa et al., 2010; Baldo et al., 2013; Renaud, 2012; Baldo et al., 2014; Grosse et al., 2017; Arrol et al., 2011; Ahn et al., 2013; Jelin et al., 2017; Guillén-Navarro et al., 2013; Mendez et al., 2015; Nicolas-Jilwan y AlSayed, 2018; Liang y Singhal, 2016; Ganesh et al., 2017; Gera, 2018; Valayannopoulos, 2015; Kubaski et al., 2020; Kubaski, 2017; Zhao, 2010; Zheng et al., 2013; Wang y Sun, 2013 [82–113]
Kinoshita M et al. 2014 Bonanni P et al., 2012 [30,31]	(6) Evaluation of the effects of ERT in epileptogenic symptoms	Scarpa et al., 2017 Bonanni et al., 2014 [57,58] (Case report)	van der Lee et al., 2017; Janzen et al., 2017; Kasteleijn- Nolst Trenite et al., 2017; Kasteleijn- Nolst Trenite, 2017; Brandl, 2014; Scarpa y Cinzia, 2013; Porter, 2017; Huang H 2013 [114–121]
Sanchez JI et al. 2015 Lau HA et al. 2015 [32,33]	7) ERT effects in vision	Yamanishi R et al. 2019 [59] (Case report)	Chen et al., 2018 [122]
Fisher et al. 2015 Uz B et al., 2012 [34,35]	(8) ERT effect in autoimmune anemia, thrombocytopenia, or thrombocitopenic purpura	Alcántara-Ortigoza et al., 2016 [60] (Case report)	Emel, 2020; Panigrahi et al., 2019 [123,124]
Nava E et al. 2012 [36]	(9) Botulinum Toxin for the treatment of equinus deformity with an ERT	New citations, but not new patient data	Kuzenkova et al., 2012; Pawliuk et al., 2020 [125,126]

Farooq MU et al., 2008 [37]	(10) ERT effects in involuntary movements (chorea)	New citations, but not new patient data	Guo et al., 2009 [127]
Papadia F et al., 2011 [38]	(11) Early ERT effects in bone abnormalities	Manara R et al. 2011 ** [53] (Cohort study)	Christianto et al., 2013; Tanaka et al., 2012; Higuchi et al., 2012; Guillén-Navarro et al., 2013; van der Lee et al., 2017; Janzen et al., 2017; Coutinho et al., 2012; Noh y Lee, 2014; Tanaka et al., 2018; Zuber et al., 2015; Rout-Pitt, 2015 [41,56,89,102,114,115,128–132]

ERT—enzyme replacement therapy, MPS-II—mucopolysaccharidosis type II. * Some case reports selected in meta-analysis are been reported in this table. They analyzed similar objectives described in previous randomized and nonrandomized studies (Muenzer et al. 2006, Muenzer et al. 2010, Tolar J. et al. 2008, Wynn F. et al. 2009, and Eisengart J.B. et al. 2013 [16–20]). The case reports Lampe et al. 2014, Bivina et al. 2014, Christiano et al. 2013, Sato et al. 2013, Tajima et al. 2013, Hoffmann B. et al. 2011, Tylki-Szymanska et al. 2012, Pérez-Calvo et al. 2011, Tchan M.C. et al. 2011, Westhoff M. et al. 201,1 and Galán Gómez E. et al. 2008 [39–49]. ** This study analyzed two novelties proposed by previous case reports. References are included in main manuscript.
